# Costs and cost-effectiveness of a large-scale mass testing and treatment intervention for malaria in Southern Province, Zambia

**DOI:** 10.1186/s12936-015-0722-3

**Published:** 2015-05-20

**Authors:** Kafula Silumbe, Joshua O Yukich, Busiku Hamainza, Adam Bennett, Duncan Earle, Mulakwa Kamuliwo, Richard W Steketee, Thomas P Eisele, John M Miller

**Affiliations:** Malaria Control and Evaluation Partnership in Africa (PATH-MACEPA), Lusaka, Zambia; Center for Applied Malaria Research and Evaluation, Tulane University School of Public Health and Tropical Medicine, New Orleans, LA USA; National Malaria Control Centre, Lusaka, Zambia; Malaria Elimination Initiative, University of California San Francisco, San Francisco, USA

**Keywords:** Malaria, Cost-effectiveness, Mass testing and treatment

## Abstract

**Background:**

A cluster, randomized, control trial of three dry-season rounds of a mass testing and treatment intervention (MTAT) using rapid diagnostic tests (RDTs) and artemether-lumefantrine (AL) was conducted in four districts in Southern Province, Zambia.

**Methods:**

Data were collected on the costs and logistics of the intervention and paired with effectiveness estimated from a community randomized control trial for the purpose of conducting a provider perspective cost-effectiveness analysis of MTAT *vs* no MTAT (Standard of Care).

**Results:**

Dry-season MTAT in this setting did not reduce malaria transmission sufficiently to permit transition to a case-investigation strategy to then pursue malaria elimination, however, the intervention did substantially reduce malaria illness and was a highly cost-effective intervention for malaria burden reduction in this moderate transmission area. The cost per RDT administered was estimated to be USD4.39 (range: USD1.62-13.96) while the cost per AL treatment administered was estimated to be USD34.74 (range: USD3.87-3,835). The net cost per disability adjusted life year averted (incremental cost-effectiveness ratio) was estimated to be USD804.

**Conclusions:**

The intervention appears to be highly cost-effective relative to World Health Organization thresholds for malaria burden reduction in Zambia as compared to no MTAT. However, it was estimated that population-wide mass drug administration is likely to be more cost-effective for burden reduction and for transmission reduction compared to MTAT.

## Background

The scale-up of vector control for malaria in sub-Saharan Africa has been extensive in the past decade [1]. In the context of this scale-up, there have been calls for malaria elimination and eradication, and increased recognition that control and elimination strategies may need to focus on the parasite reservoir in addition to reductions in human vector contact [2, 3]. The Zambian Ministry of Health (MoH) National Malaria Control Centre (NMCC), in collaboration with partners, has set high targets for malaria control intervention coverage and reductions in malaria burden, as outlined in the National Malaria Strategic Plan [4]. There is now empirical evidence of progress in rolling out malaria interventions to affected communities and their effectiveness in reducing the malaria burden in Zambia [5–11]. The Malaria Indicator Surveys conducted in 2006–2012 have shown substantial progress in making malaria control services widely available in Zambia, including prompt effective case management, possession and use of insecticide-treated mosquito nets (ITNs), availability of indoor residual spraying (IRS), and intermittent preventive treatment (IPTp) for pregnant women. Further, these surveys showed a reduction of national malaria parasite prevalence and severe anaemia among children under five years of age [5, 9, 10]. While these results suggest Zambia is moving towards measurable health impact as a result of scaled malaria interventions, they also suggest that even with high coverage of vector control and diagnosis and treatment, additional steps are necessary to continue to reduce the malaria burden among those at risk.

For these reasons the NMCC decided to embark on a large-scale trial, in the context of sustaining high vector control coverage, of three dry-season rounds of a mass testing and treatment intervention (MTAT) aimed at reducing the parasite reservoir in humans in southern Zambia as well as possibly interrupting transmission [12]. The MTAT strategy used rapid diagnostic tests (RDTs) to identify individuals with parasite infections in the community and treated those with a positive RDT with artemether-lumefantrine (AL). While mass drug administration (MDA) (the administration of an anti-malarial drug at therapeutic doses to an entire population) is known to at least temporarily reduce the burden of malaria in some settings, less is known about the effectiveness of MTAT campaigns [13]. Dynamic, deterministic, mathematical, malaria modelling indicates that it should increase the proportion of actual infections that receive effective treatment and thereby reduce the burden of clinical malaria as well as reduce the parasite reservoir with the possibility of eventually interrupting transmission [14]. In contrast, recent micro-simulation models indicate that such an approach is unlikely to succeed in interrupting transmission but may be a cost-effective way to reduce the burden of malaria after vector control scale-up has been achieved but where a moderate burden remains [15]. Given the uncertainty surrounding model inputs there is need to verify the effectiveness of these strategies in robust field trials. Furthermore, the modelled finding that MTAT is a potentially cost-effective burden reduction strategy in moderate burden locations hinges not only on the effectiveness estimates but also on cost and logistic estimates [15]. Unfortunately, very little information on the logistics, costs and operational constraints of MTAT strategies in sub-Saharan Africa is available in the literature from which to base either the parameterization of such cost models or to assist those involved in malaria control and elimination programmes to adequately plan for the financing and operation of these strategies. This paper presents the findings of an economic evaluation of three dry-season rounds on an MTAT intervention conducted in Southern Province, Zambia in 2012, which was conducted alongside a large-scale trial of the intervention [12]. This study compares the cost and cost-effectiveness of MTAT to Standard of Care (no MTAT) in southern Zambia, additionally it uses sensitivity analysis to explore programme modifications including mass drug administration.

## Methods

### Study sites

**Fig. 1:**
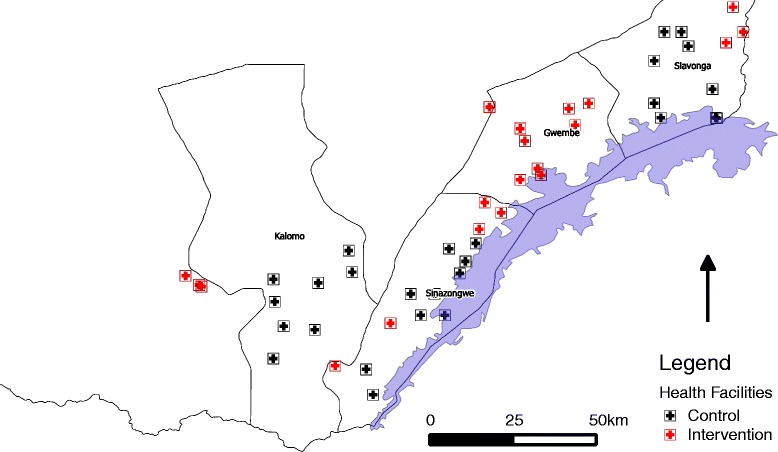
Map of Southern Province districts, health facilities and their catchment areas included in training and testing campaigns and trial

Southern Province was identified in 2010 as a study site for the MTAT trial because of its moderate malaria transmission and sustained high vector control coverage in the area. Four districts (Siavonga, Gwembe, Sinazongwe, and southeastern Kalomo) within Southern Province were selected along Lake Kariba on the southern border of Zambia and Zimbabwe (Fig. 1). The study population was estimated to be just over 330,000 people living in approximately 56,000 households in 2011.

The results of the 2006–2012 National Malaria Indicator Surveys identified Southern Province as an emerging region with lower malaria parasite prevalence among children [5, 9, 10], although facility-based reporting also suggests that much higher malaria transmission persists in districts along Lake Kariba.

### Study design

The trial utilized a cluster randomized design. Health facility catchment areas were randomly allocated to be included in the 2012 dry-season MTAT intervention arm, with control areas slotted to receive the MTAT intervention in subsequent years rolled out in a stepwise fashion. In 2012, 40 % of the health facility catchment areas included in the trial received the three dry-season MTAT rounds.

### Intervention

MTAT interventions were planned from 2011–2013 and were conducted during the dry or low malaria transmission season, which stretches from the month of May to November. The three MTAT rounds in 2012 were conducted in June-July, August-September and October-November. During each round, community health worker (CHW) teams, organized at the health facility catchment area level swept through the neighbouring community in a house-to-house fashion and tested every consenting household member using a RDT.

The systematic MTAT campaigns targeted all household members for malaria testing using RDTs within intervention catchment areas. Informed consent was obtained from all adult household members or parents or guardians of children. Each CHW was accompanied by one data assistant to capture key information about the household and its members, including questions on recent fever and treatment history, mosquito net possession, use and geo-location data. In the event that any household members with recent history of fever were not present during the household visit, or if any household members tested positive for malaria, the CHW scheduled a time to revisit the household to test these individuals. All RDT-positive individuals were treated promptly with an age/weight appropriate dose of AL according to national treatment policy guidelines. Prior to implementing MTAT campaigns, standardized training for CHWs and data assistants were conducted at a central venue within each district. The training reviewed diagnostic and treatment policies, research and consent processes as well as field practical rehearsals to evaluate and refine skill levels of field staff and supervisors.

### Cost collection and analysis

Data on intervention costs covered all of 2012. The ingredients approach was used for line item cost estimation. This means that inputs were identified, quantified, valued and classified into activity categories. Where this approach was not possible, either because the information was deemed too sensitive or was not available in adequate detail, aggregated expenditures were used or costs were estimated using WHO-CHOICE data on cost and project level data on activities [16]. Costs were classified as either capital or recurrent costs and as either traded or non-traded goods [17, 18]. In the base analysis all costs were treated as recurrent with rental costs being used to value capital goods included. Costs were initially measured in one of three currencies, Zambian Kwacha (ZMK), US dollars (USD) or International Dollars (when the source of cost information was WHO-CHOICE). As all costs were collected during the year 2012 no inflation adjustments were necessary. Costs collected in ZMK were converted to USD using official exchange rates [19]. All other costs were first converted to USD based on official yearly average exchange rates for the period during which the costs were incurred and for purchasing power parity for International Dollar costs using the World Bank purchasing power parity index [19, 20]. All costs are reported in 2012 USD. Both financial and economic costs were estimated in order to calculate the value of donated inputs as well as the actual financial implications of the intervention. Financial costs represent purely monetary flows, while economic costs represent the value (opportunity cost) of all resources necessary to implement a given intervention. However, in the case of this study no substantial donated items were used and few capital goods were used and as such the differences between financial and economic costs were negligible and only economic costs are presented here. The provider perspective was used; travel or time costs to recipients of the intervention were not included, nor were other household-level costs or cost savings. Household-level cost-savings due to averted need for malaria case management among intervention beneficiaries may have occurred. Household costs for the intervention were believed to be negligible given that the intervention is provided at no charge directly at household level and the drugs administered have very low risk of serious side effects that would require any medical intervention. Cost savings due to reduced treatment at the health facility were modelled based on existing literature on the cost of treatment of uncomplicated malaria cases at health centres in Zambia [21]. As the analysis is intended to be incremental, existing infrastructure and recurrent inputs that would be present without the intervention, such as capital costs of building health facilities or training CHWs for general roles outside this campaign, were not included.

### Logistics and output data

Data on levels of effort and outputs of the programme, including quantification of resource inputs such as artemisinin combination therapy (ACT) treatment courses, RDT kits, vehicle days, supervision days, and other programme inputs, were collected directly using programme records. Information on outputs, including the number of persons tested and the number of persons treated was collected from direct reports from CHWs administering the intervention. Coverage of the intervention was estimated using both administrative (implementers reports) data and through the individual-level data collected during the MTAT rounds, and by the use of census data collected at individual level during the intervention.

### Costing scenarios and sensitivity analysis

The base case costing scenario relied on the following set of assumptions: a discount rate of 3 % was applied to capital costs; wastage of RDT kits, ACT treatment courses and other field work materials was assumed to be 10 %; overhead for national (and international) supervision amounted to 15 % of the total direct financial costs of the programme. These are believed to be conservative assumptions. The cost of ACT treatment courses and RDT kits were based on the cost, insurance and freight (cif*.*) price of the drug or diagnostic derived from project records and the WHO Global Price Reporting Mechanism (WHO-GPRM) database [22]. Test positivity rates and population coverage estimates were assumed to be identical to those actually reported in the trial monitoring data set. In order to examine the role of these assumptions in the ultimate cost estimates, all of these parameters were varied in sensitivity analyses, the results of which are detailed in Table 1. Additionally, probabilistic sensitivity analysis was used to conduct to include uncertainty around effect and cost estimates. Catchment area level cost data was used to estimate the parameters of the distribution of cost estimates and uncertainty around effect estimates was derived from the trial results [12]. The parameters around these estimates are detailed in Table 2.

**Table 1:** Results of one-way sensitivity analysis and scenario analyses

Parameter	Base value	Sensitivity analysis value	Results/implications	Justification
Discount rate	3 %	0-10 %	No effect/All costs treated as recurrent	Covers all likely discount rate applications [45]
Cost per ACT treatment	USD 1.25	Increased to USD 4	Cost per test administered rises from USD 4.39 to 4.83	Highest value found in WHO GPRM database for an adult dose
Cost per RDT	USD 0.47	Increased to USD 2	Cost per test administered rises from USD 4.39 to 6.32	Highest cost found in WHO GPRM database
Cost per rented vehicle day	USD 208	Decreased to USD 20 per day	Cost per test administered falls from USD 4.39 to 3.65	Approximate daily capital cost of a vehicle based on WHO-CHOICE data
Use of enumerators	Included for training and field work	Excluded for training and field work	Cost per test administered falls from USD 4.39 to 3.60	Enumerators contribute substantially to research component and may be unnecessary for intervention only
NGO supervision costs	Included	Excluded	Cost per test administered falls from USD4.39 to 3.66	Ongoing NGO and international supervision may not be required if made a routine intervention
Duration of intervention effect	6 months	Reduced to 3 months/Increased to one year	Gross cost per case averted rises from USD73 to 145/Gross cost per case averted falls from USD 73 to USD 36	Duration of effect uncertain in effect analysis [12, 14, 15]

**Table 2:** Parameter inputs to probabilistic sensitivity analysis

Parameter	Input parameter value or distribution	Justification
Incidence	Poisson (lambda = 19.2)	[12]
Case fatality Rate	0.0045	[44]
Cost per person tested	Log-normal (mean log = 1.61, sd log = 0.38)	Derived from source data
DALYs per death	33	[16]
Effect size	Gamma (shape = 38.65, rate = 46.01)	[12]
DALYs per uncomplicated malaria case	0.02	[28]
Cost per case for management of uncomplicated case at a health facility	USD 6.12	[21]

### Outcome indicators

Several indicators of outcomes were calculated. These were: 1) cost per person targeted, calculated as the total costs of the intervention divided by the total number of persons targeted to receive the intervention; 2) cost per test administered, calculated as the total costs of the intervention divided by the total number of tests actually administered; and, 3) cost per treatment administered, calculated as the total costs of the intervention divided by the number of treatments administered. All outcome indicators were disaggregated to the district and health facility catchment area levels in order to facilitate sub-analysis of district level factors and health facility catchment area level factors associated with the cost-outcome relationship.

### Impact indicators

Several impact indicators were calculated: 1) cost per case averted calculated as the total costs of the intervention divided by the estimated number of malaria cases averted after exposure to three rounds of MTAT from the results of a regression model used to estimate the effects of the intervention in an accompanying paper [12]; 2) costs per death averted, calculated as the total costs of the intervention divided by the number of deaths averted for the entire targeted population based on the incident rate ratio estimated for exposure to the intervention over three rounds in a Poisson regression model, based on passive malaria data collection in the health facilities in the trial in an accompanying paper and an assumed case fatality rate and duration of effect (CFR) [12, 23–25]; and, 3) cost per disability adjusted life year (DALY) averted calculated based on the total cost of the intervention and the estimated numbers of DALYs averted for the entire population of the intervention area. DALYs averted were calculated based on the total number of malaria cases and deaths averted using Global Burden of Disease disability weights and assuming that all deaths were among under-five children and resulted in approximately 33 DALYs lost [26–28]. The study and reporting was checked against the consolidated health economic evaluation reporting standards (CHEERS) checklist for reporting of economic evaluations [29].

## Results

During the three dry-season rounds of the 2012 MTAT, a total of 269,668 tests were administered to an estimated population of 135,649 in 18 health facility catchment areas. Based on the results of the RDTs used, 34,056 people were treated for *Plasmodium falciparum* infections with AL, corresponding to a test positivity rate (TPR) of 12.6 %. Overall, average coverage of the estimated population during each round according to administrative data was 66.2 %, while coverage estimated using census data was 88.3 %; detailed results on coverage, treatment and testing by district and round are shown in Table 3. Of the 21 facilities that were included in the initial round, three facilities were discontinued during rounds two and three leaving the total intervention areas at 18. These excluded facilities were not included in the cost analysis.

**Table 3:** Outputs of the mass testing and treatment intervention by round and district

Dis	Total Pop		Round 1					Round 2					Round 3					Total			
		Test	Treat	Cov (Adm)	Cov (Cen)	TPR (Adm)	Test	Treat	Cov (Adm)	Cov (Cen)	TPR	Test	Treat	Cov (Adm)	Cov (Cen)	TPR	Test	Treat	Cov (Adm)	Cov (Cen)	TPR
Gw	57,495	45,605	10,912	79.3 %	82.5 %	23.9 %	37,837	5,031	65.8 %	85.1 %	13.3 %	37,552	4,110	65.3 %	83.8 %	10.9 %	120,994	20,053	70.1 %	83.8 %	16.6 %
Kal	11,000	5,990	300	54.5 %	81.6 %	5.0 %	6,509	262	59.2 %	96.1 %	4.0 %	6,815	150	62.0 %	97.0 %	2.2 %	19,314	712	58.5 %	89.2 %	3.7 %
Sia	45,154	24,133	1,043	53.4 %	88.8 %	4.3 %	27,284	512	60.4 %	94.0 %	1.9 %	25,151	355	55.7 %	92.5 %	1.4 %	76,568	1,910	56.5 %	91.6 %	2.5 %
Sin	22,000	18,174	5,267	82.6 %	94.8 %	2.9 %	17,110	3,660	77.8 %	96.8 %	2.1 %	17,508	2,463	79.6 %	89.4 %	1.4 %	52,792	11,390	80.0 %	93.6 %	2.2 %
Tot	135,649	93,902	17,522	69.2 %	86.2 %	18.7 %	88,740	9,465	65.4 %	90.7 %	10.7 %	87,026	7,078	64.2 %	88.2 %	8.1 %	269,668	34,065	66.3 %	88.3 %	12.6 %

*Gw* Gwembe District, *Kal* Kalomo District, *Sia* Siavonga District, *Sin* Sinazongwe District

**Fig. 2:**
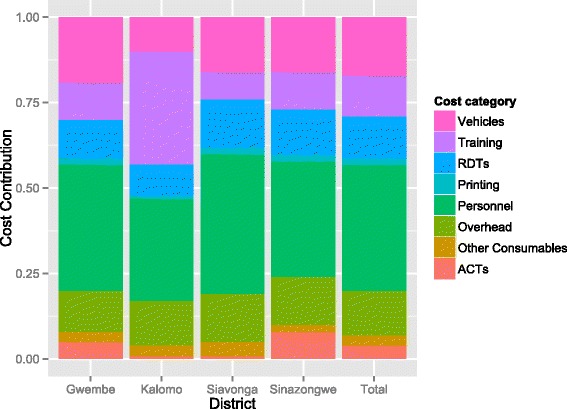
Distribution of costs by district

Total costs of the MTAT were approximately 1.2 million USD and did not vary meaningfully by round but did by district (Table 4), reflecting the large variation in included populations by district, as shown in Table 3. When costs are broken down by activity category the largest cost drivers were personnel and vehicles, with training and RDTs second (Table 5 and Fig. 2).

**Table 4:** Total costs of the mass testing and treatment campaigns by district and round

District	Round 1	Round 2	Round 3	Total
Gwembe	$ 208,993.65	$ 193,657.50	$ 191,981.78	$ 594,632.93
Kalomo	$ 35,571.77	$ 35,914.88	$ 35,970.67	$ 107,457.32
Siavonga	$ 92,632.91	$ 92,241.21	$ 92,335.87	$ 279,209.99
Sinazongwe	$ 70,447.44	$ 66,727.51	$ 65,112.41	$ 202,287.36
Total	$ 407,645.76	$ 390,541.10	$ 385,400.73	$ 1,183,587.59

**Table 5:** Total costs by activity category

Activity category	Total cost	Contribution
Training	$ 144,136	12 %
Printing	$ 23,959	2 %
Personnel	$ 435,066	37 %
Vehicles	$ 199,964	17 %
ACT	$ 46,993	4 %
RDTs	$ 139,260	12 %
Other consumables	$ 39,828	3 %
Overhead	$ 154,381	13 %
Total	$ 1,029,207	100 %

The overall cost per test administered was USD4.39, while the overall cost per treatment administered was USD34.74 (Table 6). RDTs themselves were estimated to cost USD0.47 per kit and ACT was estimated to cost USD1 for a child and USD1.25 for an adult, based on project record data. Costs per test administered varied from USD3.45 to 5.94 when summarized by district and round; the range was wider when summarized by catchment area (USD1.62-13.96). Costs per treatment administered were substantially higher, with an overall estimate of USD34.74. Costs per treatment administered varied widely when summarized by district and round (USD13.38-260.10) and even more widely when summarized by catchment area (USD3.87-3,835). Costs per treatment administered generally rose from round one through round three reflecting the declining RDT positivity rates and the subsequent need for treatment, while no clear pattern was apparent by round in cost per test administered.

**Table 6:** Economic cost per output of the mass testing and treatment (MTAT) campaigns in Southern Province, Zambia by district and round

	MTAT Round	Gwembe	Kalomo	Siavonga	Sinazongwe	Total
Cost per test administered	Rd 1	$ 4.58	$ 5.94	$ 3.84	$ 3.88	$ 4.34
	Rd 2	$ 5.12	$ 5.52	$ 3.45	$ 3.90	$ 4.40
	Rd 3	$ 5.11	$ 5.28	$ 3.67	$ 3.72	$ 4.43
	Total	$ 4.91	$ 5.56	$ 3.65	$ 3.83	$ 4.39
Cost per treatment administered	Rd 1	$ 19.15	$ 118.57	$ 88.81	$ 13.38	$ 23.26
	Rd 2	$ 38.49	$ 137.08	$ 184.06	$ 18.23	$ 41.26
	Rd 3	$ 46.71	$ 239.80	$ 260.10	$ 26.44	$ 54.45
	Total	$ 29.65	$ 150.92	$ 146.18	$ 17.76	$ 34.74

Cost savings due to averted malaria cases at the health facility and the number of deaths and DALYs averted were estimated among the entire treatment population using the overall programme effectiveness estimates from the impact evaluation. The base case scenario results for impact and cost effectiveness are shown in Table 7. The three MTAT rounds were estimated to have prevented over 16,000 malaria cases and more than 30 deaths, resulting in a net gain of more than 1,300 DALYs in the year following the intervention. This translates to a cost-effectiveness estimate of USD894 per DALY averted. Given that Zambia’s current GDP per capita is estimated at USD1,414, and utilizing WHO-CHOICE guidelines on cost effectiveness thresholds (less than GDP per capita per DALY is considered highly cost effective (less than three times GDP per capita is considered cost effective); this indicates that the MTAT intervention as implemented in Southern Province, Zambia should be considered a highly cost-effective health intervention [16].

**Table 7:** Outcome and impact indicators for the economic evaluation of the mass testing and treatment campaigns

Outcome or impact	Total	Cost per outcome or impact
Tests administered	269,668	USD 4.39
Treatments administered	34,065	USD 34.74
Persons targeted	135,649	USD 8.73
Cases averted	16,278	Gross USD 72.71
		Net USD 65.37
Estimated deaths averted	33	Gross USD 36,356
		Net USD 32,686
DALYs averted	1,324	Gross USD 894
		Net USD 804

**Fig. 3:**
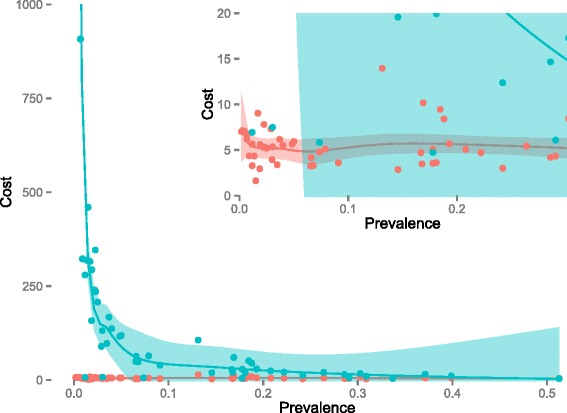
Cost per test and treatment administered *versus* prevalence. Red represents cost per test administered and green represents cost per treatment administered

**Fig. 4:**
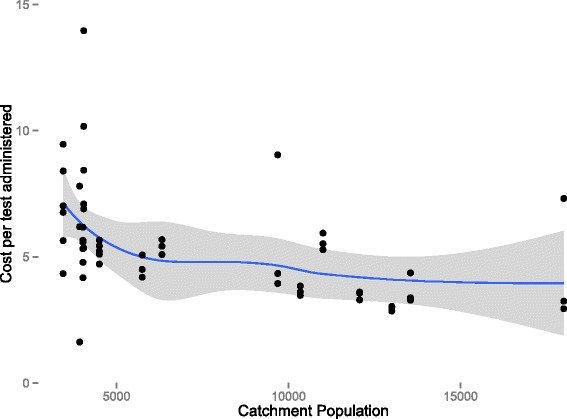
Cost per treatment administered *versus* catchment population size

Cost per test administered and cost per treatment administered varied by catchment area and round. Costs per test administered varied minimally and appeared to be independent of test positivity rate, while cost per treatment administered varied widely as previously mentioned, with a clear decreasing relationship to test positivity rate in the area during the round (Fig. 3). There appeared to be a small inverse relationship between catchment area size and the cost per person treated, although the overall magnitude is small (Fig. 4).

In order to determine the effect of various assumptions made in the analysis on the conclusions of the study, a sensitivity analysis was conducted. Sensitivity analysis indicated that the results were most sensitive to the costs of vehicles and the use of NGO staff and supervision (Table 1). None of the assumptions tested in one-way sensitivity analysis or scenario analysis altered the conclusion that the MTAT intervention was highly cost-effective as implemented in Southern Province, Zambia.

**Fig. 5:**
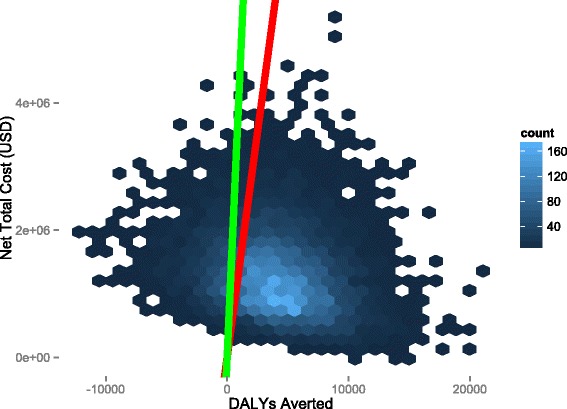
Sensitivity analysis of effect size and cost variance. Hex-bins represent simulated cost and DALY averted outcomes, with lighter colour indicating higher density of simulations, black lines are the x and y-axes of the chart. The green line is the WHO threshold for an intervention to be considered highly cost-effective in Zambia (USD 1,414 per DALY averted) and the red line is the threshold at which an intervention is considered cost-effective or not cost-effective in Zambia (3x GDP per capita) (USD 4,242 per DALY averted)

**Fig. 6:**
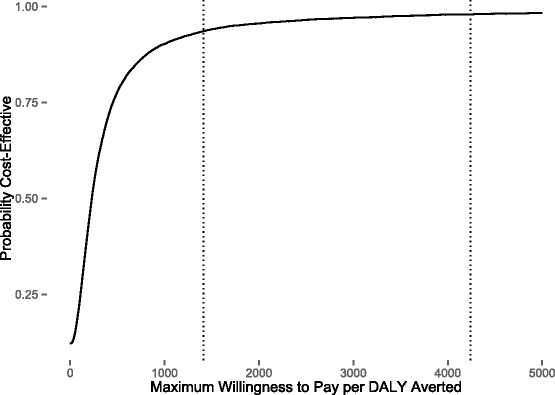
Probabilistic sensitivity analysis results shown as a cost-effectiveness acceptability curve. The vertical dotted lines represent the WHO thresholds for an intervention to be considered highly cost-effective and cost-effective in Zambia: (USD 1,414 per DALY averted) and (USD 4,242 per DALY averted), respectively

Figure 5 illustrates the results of the probabilistic sensitivity analysis of effect size and cost. The majority of simulation results lies in the first quadrant of the cost-effectiveness plane, indicating that the intervention is expected to provide additional benefits but also to require additional expenditure (Fig. 5). Detailed analysis of the results indicates that 81 % of simulations resulted in additional costs but with a cost to effect (CE) ratio, which fell below the WHO threshold for a highly cost effective-intervention. Eighty-seven per cent of simulations led to the conclusion that the intervention was at least a cost-effective intervention (WHO thresholds are illustrated by the red and blue lines in Fig. 5). Only 11 % of the simulations fell in quadrant II, indicating that the intervention would be both less effective and more costly than the alternative (in other words, MTAT was dominated by Standard of Care). The results are additionally shown in the form of a cost-effectiveness acceptability curve in Fig. 6.

## Discussion

This paper demonstrates MTAT interventions are likely to be highly cost-effective interventions for malaria burden reduction in moderate transmission areas in Zambia, but did not reduce prevalence of infection or malaria transmission significantly enough for these areas to proceed towards malaria elimination. This finding is in line with several modelling exercises, which suggest that MTAT is likely to provide burden reduction benefits in areas of moderate and high transmission and be relatively cost-effective at doing so [14, 15]. While this finding is important, significant and likely to be robust, it also needs to be taken carefully in context. Although MTAT appears highly cost effective for malaria burden reduction, the estimates reported here are more than an order of magnitude higher than those found for several other malaria control interventions, including long-lasting insecticidal nets (LLINs) (range: USD8.15-110 per DALY), IRS (range: USD135-150 per DALY) and IPTp (range: USD-329-139 per DALY) [30, 31]. As such the current findings should not be interpreted as a call to scale-up MTAT where other interventions are still neglected. One mathematical model suggests that at ITN coverage levels similar to those found in the districts where this study was conducted (>60 %), scale-up of MTAT might be similar in cost-effectiveness to further scale-up of ITNs [15]. Estimates of MTAT cost-effectiveness in this study show that MTAT would not be competitive with initial LLIN scale-up; however, the marginal cost-effectiveness of MTAT *versus* further scale-up of vector control may warrant further study.

While the study indicated the possibility that some cost savings in case management might accrue due to the implementation of MTAT, these savings appear to be modest compared to the costs of implementing the MTAT intervention. This is due to the fact that many uncomplicated malaria cases do not present at health facilities, where cost savings accrue to providers, and also to the relatively modest cost of treating an uncomplicated malaria case in the health system. This cost is very modest, especially compared to the cost per treatment administered in the very low test positivity rate areas in the study [21], indicating that, unsurprisingly, MTAT would be an extremely inefficient way to administer treatments for symptomatic infections (given the low prevalence of currently symptomatic infections in the population) in low transmission areas. In very low transmission areas, especially those with good health system access, and where a larger proportion of infections are generally expected to be symptomatic it may not be an efficient way to treat malaria infections. Even in locations where health system access is poor, investments in the improvement in health care access, such as community case management, may be more efficient at reaching infected individuals, given the massive numbers of individuals who must be tested in order to identify an infection for treatment under the MTAT intervention. Unfortunately the costs of improving health system access in relevant areas of Africa are not well understood and are understudied. MTAT may offer the advantage of reaching individuals in the general population who would never seek medical care either because of limited access or because their infections have never become significantly symptomatic for them to do so. In elimination settings MTAT might be useful even though it will require large investment to identify a small number of infected individuals. However, the effectiveness of MTAT to identify this remaining infected reservoir is also limited by the sensitivity and specificity of the diagnostics used, the actual population coverage achieved in the intervention, and the time frame and geographic area over which such an extensive investigation can be repeatedly conducted [13, 14, 32].

While this study can give no indication of the probability that interruption of transmission might occur due to repeated MTAT in the study areas, it could provide useful evidence on the cost of the use of MTAT as part of a package of interventions targeted at elimination. These data give some indication that there may be minor economies of scale in the implementation of MTAT (see Fig. 4). However, these returns appear to be modest at best.

MTAT is a variation of MDA, which has been made possible by the development of rapid, inexpensive, point of care diagnostics - malaria RDTs. However, given the results here it is clear that MDA might be a more suitable approach to the treatment of prevalent malaria infections. This is due to the fact that MDA removes the cost of RDTs, and the time associated with the testing of individuals prior to treatment. MDA is also likely to be easier to administer at community treatment points than MTAT. These two modifications could significantly reduce the costs and increase the drug coverage in the population. MDA has been shown to significantly, temporarily reduce malaria prevalence and incidence in many transmission settings in the past [13]. A trial of MDA to replace MTAT designed to measure these outcomes began in Southern Province, Zambia in 2014.

Several objections to the approach proposed above might be raised. These include: that shifting to community treatment points could lead to declines in coverage, that MDA may not be more effective than MTAT, especially if MTAT were implemented with a highly sensitive and specific diagnostic, that adherence to treatment may decline with the use of MDA as opposed to MTAT, and, that the more wide-scale use of drugs implied by MDA might create a more favourable environment for the development or spread of drug resistance [33–35].

The coverage of the MTAT intervention was estimated to be near 90 % using census data collected during the intervention, however, administrative reports coupled with health system denominators yielded much lower coverage estimates. High coverage of MTAT is likely to be highly important to successful implementation of the intervention, especially where the goal of the intervention is local elimination of the parasite reservoir. It is likely that increased coverage would have led to improved outcomes in this study and perhaps improved cost-effectiveness. However, given that coverage of the intervention may have been very high it is not likely that MTAT, at least implemented over one dry season, even with high coverage and use of AL, to interrupt malaria transmission in areas similar to Southern Province, Zambia.

Finally, little is known about treatment adherence with MTAT interventions for malaria although reported coverage in MDA interventions has varied greatly [36–41]. However, the use of a therapy that requires the user to take six doses over a period of three days is not ideal for use in either MTAT or MDA. Failure to fully complete or adhere to therapeutic recommendations for infectious diseases has been shown in numerous settings, even among symptomatic patients [42, 43]. Given both the relatively limited access to health care in this setting and the relatively limited access to resources of most of the population there is a strong likelihood that a significant portion of patients will fail to complete therapy and instead reserve the remainder of drug for a period in which they are ill.

The results of this study depend on a number of assumptions; while the sensitivity analysis conducted herein lends credence to the robustness of the results - especially their internal validity - the risk remains that neither the costs nor the effects may be generalizable. Because the DALY burden for malaria is driven largely by mortality, the assumed case fatality rate will have a large influence on cost-effectiveness outcomes. The numbers are highly uncertain and while this study uses WHO estimates, variation in this quantity could significantly alter results [44].

These results suggest that MTAT, while a highly cost-effective intervention for malaria burden reduction, is still a relatively expensive investment for malaria control and is much less cost-effective than initial roll-out of ITNs or IRS [30, 31]. Additionally, MTAT did not lead to local elimination of malaria in any part of the study area. There are, however, substantial benefits that are derived from an intervention of this type, which cannot be easily valued and included in such an analysis. Aside from potential cost-savings due to averted treatments from the household side, there are numerous benefits that were not, or could not be, included. These include the improved knowledge of household locations and parasite spatial distribution which arise when conducting household-to-household MTAT with attendant data collection and household geo-location, and the reach of the health system into communities with limited access to care.

In line with the goals of the National Malaria Strategic Plan, optimal approaches aimed at further reducing malaria infection in Zambia are needed given the existing levels of malaria prevention coverage. Implementing an MTAT campaign is a challenging and resource-intensive activity requiring significant microplanning at every level of the health system. This paper measures the costs of implementing a campaign of house-to-house MTAT for malaria. This study finds that MTAT is a highly cost-effective intervention for burden reduction in moderate transmission areas in Zambia and provides cost and logistic information which may be translatable to other settings intending to implement an intervention of a similar type.

## Conclusions

MTAT of entire populations for malaria infection is a highly cost-effective intervention for malaria burden reduction in Southern Province, Zambia. However, the reductions achieved were not sufficient for these areas to transition into case investigation or to interrupt transmission. These finding are likely to be robust within areas of moderate transmission and in other similar African settings. However, MTAT remains a less efficient way to deliver drugs to a population (compared to MDA) although it would likely result in lower drug pressure on the parasite reservoir than would occur in a MDA campaign. MTAT is less cost-effective than scale-up of the main vector control interventions and should not be seen as an alternative to scale-up of these proven vector control interventions. In very low prevalence areas, MTAT may be less efficient at delivering treatment than simple passive health system treatment of active cases, although it likely covers individuals who otherwise would not receive curative treatment.
